# Genome Sequences of Two F1 Subcluster Bacteriophages, Burwell21 and Nivrat, Isolated Using the Bacterial Host Mycobacterium smegmatis

**DOI:** 10.1128/MRA.01594-19

**Published:** 2020-03-12

**Authors:** Joanna Katsanos, Chioma Ngene, Jennifer Cook-Easterwood

**Affiliations:** a Department of Biology, Queens University of Charlotte, Charlotte, North Carolina, USA; b Department of Chemistry, Queens University of Charlotte, Charlotte, North Carolina, USA; DOE Joint Genome Institute

## Abstract

The mycobacteriophages Burwell21 and Nivrat are two F1 cluster bacteriophages isolated from different soil samples in Charlotte, NC. Burwell21 has a 58,098-base-pair double-stranded DNA genome, with 99 protein-coding genes predicted, whereas Nivrat has a 58,009-base-pair genome, with 102 protein-coding genes predicted.

## ANNOUNCEMENT

We report the genome sequences of two F1 subcluster mycobacteriophages, Burwell21 and Nivrat, that were isolated from two different soil samples (35.288°N, 80.832°W and 35.189°N, 80.834°W, respectively). Both soil samples were collected from flower beds (rich, dark soil) on the campus of Queens University of Charlotte in North Carolina as part of the Howard Hughes Medical Institute (HHMI) Science Education Alliance-Phage Hunters Advancing Genomic and Evolutionary Science (SEA-PHAGES) program (1). The isolation, characterization, genome sequencing, and annotation of Burwell21 and Nivrat contribute to a greater understanding of the diversity and abundance of mycobacteriophage species (1).

We used the protocols provided in the HHMI SEA-PHAGES discovery guide (https://seaphagesphagediscoveryguide.helpdocsonline.com/home) to isolate the mycobacteriophages by enrichment using the bacterial host Mycobacterium smegmatis mc^2^ 155 (provided by the Bacteriophage Institute at University of Pittsburgh) grown in 7H9 complete liquid medium at 37°C for 5 days. Isolation was followed by multiple rounds of plaque assays to purify the phage and the generation of high-volume lysates to amplify the phage. Subsequent negative-staining transmission electron microscopy revealed that Burwell21 and Nivrat have siphoviral morphology. Burwell21 and Nivrat both have a 200-nm tail; however, the diameters of their capsids were 67 nm and 57 nm, respectively.

Genomic DNA was isolated from the bacteriophage using the Promega Wizard DNA cleanup kit and prepared for sequencing with a NEB Ultra II DNA kit. Genomes were sequenced at the Pittsburgh Bacteriophage Institute with an Illumina MiSeq instrument, yielding at least 720,000 reads of 150-base read length per genome (Table 1). The raw reads were assembled using Newbler 2.7 (2) with default settings in each case, yielding a single contig for each phage. Consed v29 (3) was used to check for completeness, accuracy, and genomic termini, as described by Russell (4). Burwell21 had a genome size of 58,098 base pairs, with a fold coverage of 1,753×, a G+C content of 61.5%, and 10-base single-stranded 3′ extensions (5′-CGGTAGGCGC-3′). Nivrat had a genome length of 52,152 base pairs, with a fold coverage of 1,768×, and also had a G+C content of 61.5% with 10-base single-stranded 3′ extensions (5′-CGGAAGGCGC-3′).

**TABLE 1:** Phage accession numbers and genome assembly results

Phage name	GenBank accession no.	SRA accession no.	No. of reads	Avg coverage (×)	Cluster	Genome length (bp)	G+C content (%)	No. of genes
Burwell21	MH651169	SRX5572879	720,592	1,753	F1	58,098	61.5	99
Nivrat	MH651183	SRX5572868	724,515	1,768	F1	52,152	61.5	102

The protein-coding regions of the phage genomes were predicted using GLIMMER v3.02 (5) and GeneMark v2.5p (6) embedded within DNA Master v5.02 (http://cobamide2.bio.pitt.edu/computer.htm) using the settings described in the HHMI SEA-PHAGES Bioinformatics Guide (https://seaphagesbioinformatics.helpdocsonline.com/home). Gene starts were predicted using Starterator (https://seaphages.org/software/#Starterator). Protein functions were determined using NCBI BLASTp 2.7 (7), Phamerator (8), and HHPred v3.0beta (9). Burwell21 was assigned 42 putative functions to the 99 predicted protein-coding regions, whereas Nivrat was assigned 31 putative functions to the 102 protein-coding genes. No tRNA sequences were identified in either bacteriophage genome by ARAGORN v1.1 (10). Burwell21 and Nivrat both contained translational frameshifts in the tail assembly chaperone genes.

Clustal Omega alignment (11) using default settings between Burwell21 and Nivrat showed a 92.8% average nucleotide sequence identity (ANI) between them. Subsequent Clustal Omega multiple alignments using default settings indicated a 95.5% ANI between Burwell21 and Cabrinians (GenBank accession number KT895281), whereas Nivrat had a 90.4% ANI with Cabrinians. Burwell21 and Nivrat also have 85.3% and 86.1% ANI, respectively, with Saal (GenBank accession number KJ025956). Cabrinians and Saal are both members of the F1 subcluster; hence, Burwell21 and Nivrat are also members of the F1 mycobacteriophage subcluster.

### Data availability.

The GenBank and SRA accession numbers for Burwell21 and Nivrat are listed in Table 1.
